# Oxidative Damage to RNA is Altered by the Presence of Interacting Proteins or Modified Nucleosides

**DOI:** 10.3389/fmolb.2021.697149

**Published:** 2021-07-01

**Authors:** Mariana Estevez, Satenik Valesyan, Manasses Jora, Patrick A. Limbach, Balasubrahmanyam Addepalli

**Affiliations:** Rieveschl Laboratories for Mass Spectrometry, Department of Chemistry, University of Cincinnati, Cincinnati, OH, United States

**Keywords:** oxidative stress, post-transcriptional modifications, RNA damage, 8-oxo-G, ribosomes, mnm ^5^s^2^U

## Abstract

Oxidative stress triggered by the Fenton reaction (chemical) or UVR exposure (photo) can damage cellular biomolecules including RNA through oxidation of nucleotides. Besides such xenobiotic chemical modifications, RNA also contains several post-transcriptional nucleoside modifications that are installed by enzymes to modulate structure, RNA-protein interactions, and biochemical functions. We examined the extent of oxidative damage to naturally modified RNA which is required for cellular protein synthesis under two different contexts. The extent of oxidative damage is higher when RNA is not associated with proteins, but the degree of damage is lower when the RNA is presented in the form of a ribonucleoprotein complex, such as an intact ribosome. Our studies also indicate that absence of methylations in ribosomal RNA at specific positions could make it more susceptible to photooxidative stress. However, the extent of guanosine oxidation varied with the position at which the modification is deficient, indicating position-dependent structural effects. Further, an *E. coli* strain deficient in 5-methylaminomethyl-2-thiouridine (mnm^5^s^2^U) (found in lysine and glutamate tRNA anticodon) is more vulnerable to oxidative RNA damage compared to its wildtype strain suggesting an auxiliary function for the mnm^5^s^2^U modification. These studies indicate that oxidative damage to RNA is altered by the presence of enzymatic modified nucleosides or protein association inside the cell.

## 1 Introduction

Disruption of the prooxidant-antioxidant balance in favor of prooxidant leads to elevated levels of reactive oxygen species (ROS) and oxidative stress. ROS include superoxide (O˙_2_^-^) anion, hydroxyl radical (˙OH), singlet oxygen (^1^O_2_), and hydroperoxyl radical (HOO˙) generated by incomplete reduction of oxygen by mitochondrial dysfunction (Lin and Beal, 2006; Moreira et al., 2007), Fenton reaction (Koppenol, 1993), UV light exposure (Schuch et al., 2017; Yagura et al., 2017) or pollutants (Leni et al., 2020; Maher et al., 2020). While transition metal ions like iron (II), copper and hydrogen peroxide (H_2_O_2_) initiate Fenton reaction (Koppenol, 1993, Koppenol, 2001), solar UV radiation with its 95% UVA (λ 315–400 nm) component produces ROS through interaction with photosensitizers (Cadet et al., 2009; Yagura et al., 2017) (Scheme 1). The highly reactive ROS causes unspecific oxidation of proteins (Chondrogianni et al., 2014), lipids (Mylonas and Kouretas, 1999), and nucleic acids (Wiseman and Halliwell, 1996; Li et al., 2006). Cells contain exquisite but conserved DNA repair pathways to avoid permanent damage to genetic material, while other biomolecules are expected to be recycled (Huang et al., 2020; Hussain et al., 2020).

RNA is the most abundant nucleic acid in the cell. Its amount is ∼4 times more than DNA and exhibits 10–20 times higher ROS-mediated damage due to its subcellular distribution (Nunomura et al., 1999; Shen et al., 2000; Hofer et al., 2005). Oxidative damage to RNA was ignored for a long time as it is assumed to be a transient messenger of DNA. However, the linkage of oxidative RNA damage prior to protein and lipid damage in Alzheimer’s disease (Nunomura et al., 1999) and other aging-related diseases (Liguori et al., 2018; Li et al., 2020) lead to renewed interest in this field. The aggravated damage is due to the longer half-life of neuronal mRNAs (∼10 h) (Yang et al., 2003) and noncoding RNAs (Defoiche et al., 2009) leading to adverse effects on protein expression unless the damaged RNA is cleared (Ishii and Sekiguchi, 2019; Li et al., 2020). The reasons for higher damage to RNA include single-stranded nature, transitory protein binding and lack of repair (Li et al., 2006; Küpfer and Leumann, 2014).

Although all four nucleosides of RNA are affected by ROS, guanosine is more vulnerable to oxidative damage due to its low redox potential (1.29 V vs NHE) compared to adenosine (1.42 V vs NHE) and pyrimidines (>1.6 V vs NHE) (Steenken and Jovanovic, 1997; Küpfer and Leumann, 2014). Indeed, 8-oxoguanosine (8-oxoG) is the most ubiquitous oxidation product formed either in DNA or RNA (Wurtmann and Wolin, 2009; Cadet et al., 2016). Hydrogen peroxide (H_2_O_2_)-treated *Escherichia coli* cells exhibited ∼5-fold increase in 8-oxoG levels in RNA compared to DNA and is widely distributed in ribosomal and non-ribosomal RNAs as detected by HPLC analysis (Liu et al., 2012). Oxidized nucleotides at specific sites of the ribosome catalytic center inhibited the elongation cycle of protein synthesis (Willi et al., 2018) and caused errors during codon-anticodon pairing in the translation process (Taddei et al., 1997; Tanaka et al., 2007; Poulsen et al., 2012). H_2_O_2_-induced oxidative stress also inactivates tRNA^Gly^ thereby reducing translational efficiency (Leiva et al., 2020). Apart from 8-oxoG, ROS exposure causes multiple oxidative products in DNA and RNA (Barciszewski et al., 1999) and their characterization requires additional analytical approaches.

Liquid chromatography coupled with mass spectrometry (LC-MS) is an ideal platform for documenting the identity of oxidation products based on oxidation-induced alterations in mass values. This analytical platform is extensively used for comprehensive and unbiased detection and identification of xenobiotic or natural post-transcriptional modifications (PTMs) (Jora et al., 2019). The naturally occurring PTMs impart structural stability (Decatur and Fournier, 2002; Väre et al., 2017), facilitate interactions between ribosomal RNA (rRNA), transfer RNA (tRNA), messenger RNA (mRNA) and translation factors (Polikanov et al., 2015), and impart translation efficiency (Agris et al., 2017). Our laboratory has previously documented oxidative changes to ribonucleosides and their PTMs in *E. coli* upon UVA-induced oxidative stress (Sun et al., 2018) and these oxidative changes seem to localize at specific regions of tRNA (Barciszewski et al., 1999; Sun et al., 2020). Here, we show that the oxidative changes in RNA are altered by the bound proteins and post-transcriptional nucleoside modifications such as methylations or the hypermodification, 5-methylaminomethyl-2-thiouridine (mnm^5^s^2^U), found in ribosomal RNA and tRNA, respectively, of an *E. coli* model system.

**SCHEME I:**
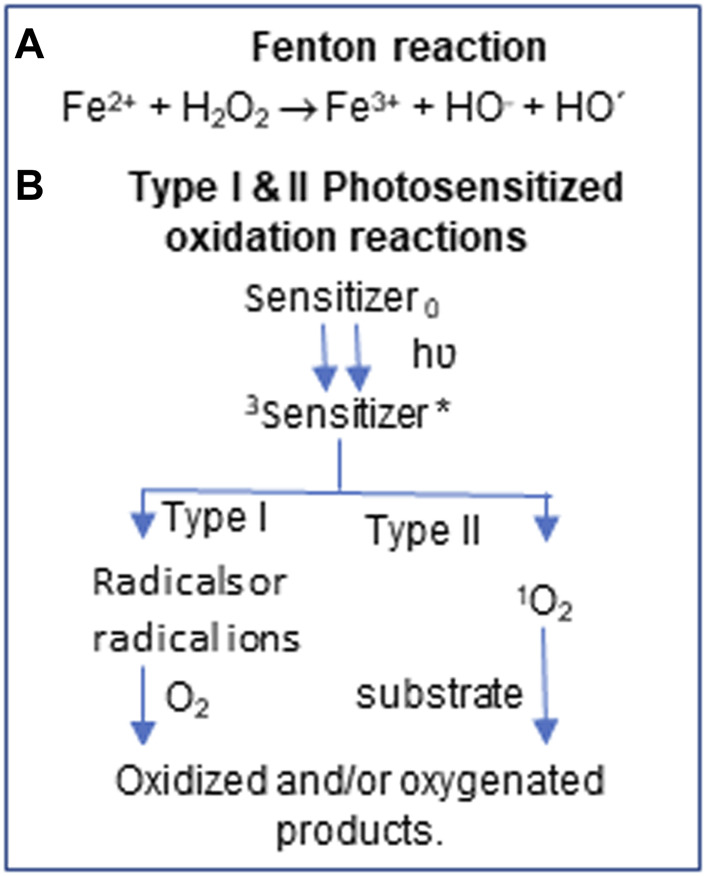
ROS generation by Chemical and photooxidation. ^3^Triplet state *highly reactive state

## 2 Materials and Methods

*E. coli* strains were obtained from the *E. coli* Genetic stock center (cgsc.biology.yale.edu). Iron chloride (FeCl_2_), riboflavin, H_2_O_2_ and all other chemicals were procured from Fisher Scientific (Fairlawn, NJ, United States) unless otherwise specified.

### 2.1 Ribosome Isolation

*E. coli* strains listed in Table 1 were cultured in LB media to ∼0.5 U (OD_600_) and harvested by centrifugation. The pellet was resuspended in lysis buffer (20 mM Tris-HCl pH 7.5, 100 mM NH_4_Cl, 0.5 mM EDTA, 6 mM β-mercaptoethanol). Lysozyme was added (1.5 mg/ml) to the suspension and incubated on ice for 10 min and the cell lysate centrifuged (30,000xg, 15 min, at 4°C). After incubation of the supernatant for five more min with 20 U of RQ1 DNase on ice, the suspension was re-centrifuged for 15 min at 30,000xg. The supernatant was layered on top of an equal volume of 32% sucrose solution and subject to centrifugation at 100,000xg for 18 h at 4°C to pellet the ribosomes (Mehta et al., 2012). The ribosomal pellet was resuspended in ribosome storage buffer (50 mM Tris-HCl pH 7.5, 100 mM NH_4_Cl, 10 mM MgCl_2_, 6 mM β-mercaptoethanol) and stored at −80 °C until use.

**TABLE 1:** *E. coli* strains used for oxidative exposure studies of ribosomes and rRNA.

Name	Strain	Genotype	Source
WT (K12)	K12 MG-1655	F-, **λ** ^*-*^, *rph-1*	ATCC 47076
*ΔrlmKL*	JW0931-1	F-, *Δ(araD-araB)567*, *ΔlacZ4787* (::rrnB-3), *λ* ^*-*^, *ΔrlmL764::kan*, *rph-1*, *Δ*(*rhaD-rhaB*)*568*, *hsdR514*	CGSC Baba et al. (2006)
*ΔrlmB*	JW4138-1	F-, *Δ(araD-araB)567*, *ΔlacZ4787* (::rrnB-3), *λ* ^*-*^, *rph-1*, *Δ*(*rhaD-rhaB*)*568*, *ΔrlmB730::kan*, *hsdR514*	CGSC Baba et al. (2006)
*ΔtrmU*	JW1119-1	F-, *Δ(araD-araB)567*, *ΔlacZ4787* (::rrnB-3), *λ* ^*-*^, *ΔtrmU721::kan*, *rph-1*, *Δ*(*rhaD-rhaB*)*568*, *hsdR514*	CGSC Baba et al. (2006)

### 2.2 Ribonucleic Acid Isolation

To obtain protein-free ribosomal RNA, ribosomes were extracted with Tri-Reagent® (Sigma-Aldrich) as per the manufacturer’s protocol. Ribosomes and ribosomal RNA were stored at −80°C.

### 2.3 UVA and H_2_O_2_ Exposures

Ribosomes (30 μg; 1A260 = 60 µg) and rRNA (7 μg; 1A260 = 40 µg) in storage buffer (0.12 μg μl^−1^) were irradiated with UVA (370 nm peak wavelength, 360–400 nm range, 0.752 mJ cm^−2^) at room temperature (25°C) for 1 h with addition of 100 µM riboflavin (RF). Unexposed samples were kept in the dark, under the same conditions. Another set of ribosomes and rRNA samples were also incubated with 10 µM iron (II) chloride and 20 mM H_2_O_2_ for 1 h at 37°C. Unexposed samples were treated similarly but no H_2_O_2_ was added. Soon after exposure, rRNA was extracted from ribosomes using Tri-reagent. RNA was reprecipitated with ½ volume of 7.5 M ammonium acetate and three times volume of ethanol, pelleted, and dissolved in sterile water.

For in-cellular exposure, cells were grown to 0.5 OD_600 nm_ and exposed to 10 µM FeCl_2_ and 20 mM H_2_O_2_ for 30 min (Willi et al., 2018) or UVA for a defined period of time, RNA extracted and ribosomal RNA fractionated by lithium chloride-based precipitation with no lag time (Nilsen, 2012).

### 2.4 Ribonucleic Acid Quality Analysis

The quality of isolated rRNA was analyzed by the Fragment Analyzer (Advanced Analytical) at Cincinnati Children core facility. The vendor-specific software (ProSize) was used to compute the signal intensity for 23S rRNA and 16S rRNA following gel electrophoresis.

### 2.5 Monitoring *E. coli* Growth Curves

*E. coli* strains, K12 (wild type), Δ*rlmKL* and Δ*rlmB* were grown to mid-log phase (∼0.5 OD600 nm) and diluted to ∼0.01 OD for testing growth behavior when grown with LB media. Growth was monitored through changes in OD using a microplate reader (Biotek 80 TS) at 37°C with slowest shake speed setting for 24 h.

### 2.6 Nucleoside Analysis

Four µg of UVA or H_2_O_2_-exposed and unexposed RNA samples were digested to nucleosides following denaturation (heating at 90°C, 10 min, snap cooling on ice) and incubated with 0.3 U of nuclease P1 (Sigma-Aldrich) in 10 mM ammonium acetate at 45°C for 2 h. To this digest, ammonium bicarbonate (100 mM), 0.00125 U of snake venom phosphodiesterase and 0.1 U of bacterial alkaline phosphatase were added and incubated at 37°C for 2 h. Digests were dried and resuspended in mobile phase A (MPA) (5.3 mM ammonium acetate, pH 4.5) before subjecting it to LC-MS. A Vanquish UHPLC coupled with an Orbitrap Fusion Lumos (Thermo Scientific) or TSQ Quantiva Triple Quadrupole Mass spectrometer were used for data acquisition. Reversed phase liquid chromatography was performed using an HSS T3 column (100 Å, 1.8 μm, 2.1 × 50 mm, Waters) and the mass spectra were recorded in positive polarity. LC-MS/MS conditions were identical to those described previously (Jora et al., 2018). Nucleosides were identified based on chromatographic retention time, unique *m/z* values, and CID/HCD fragmentation. The data was processed using Xcalibur software (Thermo Fisher Scientific) for subsequent interpretation.

## 3 Results

### 3.1 The Type of Oxidative Lesions in RNA Depends on the Kind of Oxidative Stress and Association With Proteins

Purine nucleobases undergo oxidative damage to form oxidation products just like DNA (Scheme II). However, the prevalence of such products is not clear when RNA is bound with proteins. To obtain more insight into these events, we subjected the intact ribosomes or purified ribosomal RNA to UVA or Fenton-reaction induced oxidative stress and documented the type of oxidation products observed for each condition. The LC-MS technique employs electrospray ionization to convert the ions in the liquid phase to gaseous phase at the entrance (source) of mass spectrometer. Such a process, in general, causes oxidation of the analyte at the source. Guanosine with its low redox potential is more susceptible to such oxidation. However, such an electrospray-induced oxidation can be easily distinguished as its signal aligns identically to that of the chromatographic retention time of unoxidized nucleoside (data not shown). On the other hand, the native 8-oxo-G (present in nucleoside mix prior to electrospray) exhibited a longer retention time on the chromatographic column and generates a separate signal enabling its distinction (Supplemental Figure S1A).

**SCHEME II:**
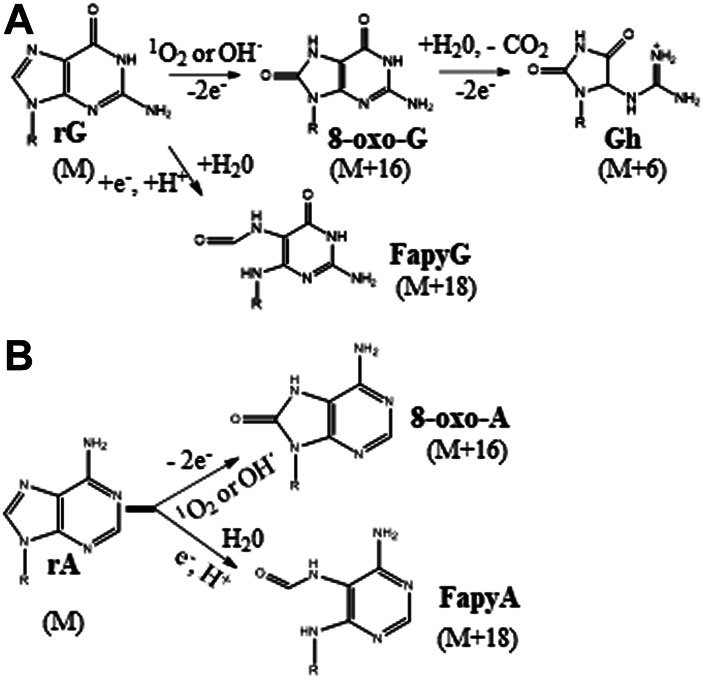
**(A)** Guanosine oxidation by the reactive oxygen species. The oxidation products, 8-oxo-G (8-oxoguanosine), Gh (guanidinohydantoin), and FapyG (2,6-diamino-4-hydroxy-5-formamidopyrimidine) and the change in molecular mass (M) of guanosine in parenthesis are shown. **(B)** Adenosine oxidation by the reactive oxygen species. The oxidation products, 8-oxo-A (8-oxoadenosine), and FapyA (4,6-diamino-5-formamidopyrimidine) are shown. These schemes are adopted from Cadet et al. (2016).

We noticed the presence of 8-oxo-G (*m/z* 300.0944) lesions in unexposed rRNA at an average of ∼one to two for every 1,000 guanosines when cells were harvested at mid-log phase (Supplementary Figure S1B). When RNA was incubated with either FeCl_2_ or RF without H_2_O_2_ or UVA exposure, respectively, variability at the basal levels of 8-oxo-G was observed. So, in order to account for these effects, changes in 8-oxo-G levels are expressed as fold change between the oxidant included and omitted samples but contained either FeCl_2_ or RF in the respective sets. However, advanced oxidation product (further oxidation of 8-oxo-G) levels were at baseline levels in the oxidant omitted samples. In these cases, the observed peak areas of oxidation products were normalized against defined number of guanosines (10^3^ or 10^5^).

**FIGURE 1:**
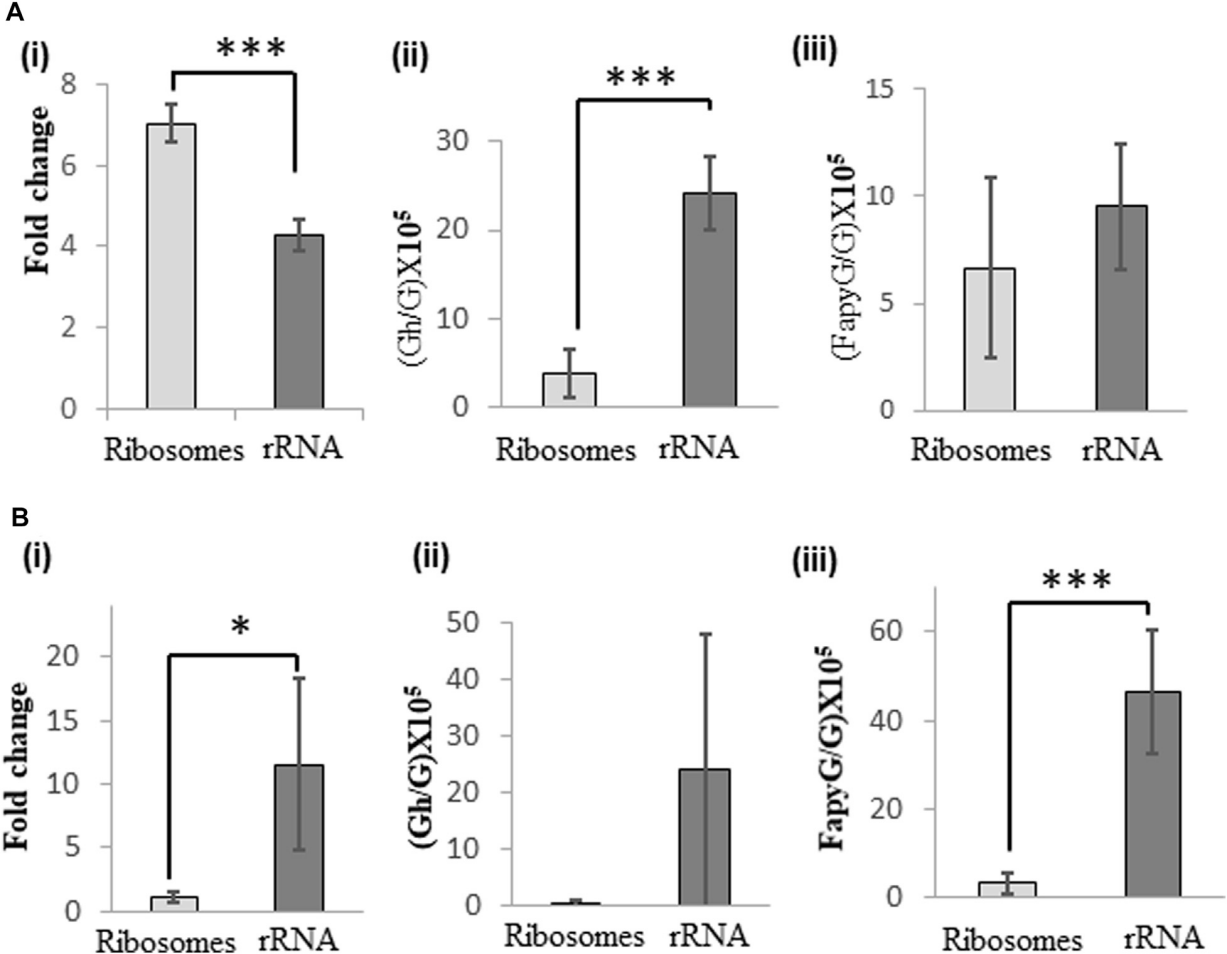
Changes in levels of guanosine oxidation products of ribosomal RNA following exposure of ribosomes and rRNA to oxidative stress. **(A)** UVA-induced changes in levels of (i) 8-oxo-G, (ii) Gh, and (iii) FapyG in rRNA and ribosomes. **(B)** Fenton-reaction induced changes in levels of (i) 8-oxo-G, (ii) Gh and (iii) FapyG upon exposure of rRNA or ribosomes. To account for the oxidation products detected in unexposed samples, Fold Change between exposed/unexposed samples was computed following normalization of the quantum of each signal against internal standard 5′bromo-2′deoxycytidine. Gh and FapyG levels are expressed as per 10^5^ G, as they were not detected in unexposed samples. Data are shown as the mean of five independent samples ±SD. Statistical differences were determined using unpaired two-tailed *t*-test (*p < 0.05; ***p < 0.001).

*Guanosine oxidation products*: Less than 60 min of UVA exposure of ribosomes did not cause significant increase in 8-oxo-G levels and more than 60 min exposure resulted in fragmentation of RNA (data not shown). Therefore, 60 min was chosen as the optimal exposure time to differentiate the oxidative effects due to protein presence in the ribosome. Upon UVA exposure we see higher levels of 8-oxo-G and guanidinohydantoin (Gh) (*m/z* 290.1097) lesions (Supplementary Figure S2A) compared to the unexposed (but riboflavin-containing) samples. However, the UVA-induced differences show contrasting features between ribosomes and rRNA even though they were exposed under identical buffer conditions. While the 8-oxo-G levels were significantly higher for ribosomes (7-fold vs 4-fold for rRNA), Gh levels were at increased levels for rRNA, 24) compared to ribosomes (4 per 10^5^ G). This suggests that association with proteins could potentially decrease the chances of further oxidation of 8-oxo-G in the ribosomes. Lack of associated proteins presumably enabled advanced oxidation to Gh (4-electron oxidation product) in protein-free rRNA. Interestingly, the observed levels for FapyG (*m/z* 302.1101) (Supplementary Figure S2B) were lower (7–9 per 10^5^ G) upon UVA exposure and do not show significant differences between ribosome and rRNA samples suggesting that this type of alternate oxidation process occurs at relatively low level irrespective of the protein association status (Figure 1A).

Fenton reaction-induced oxidative stress, on the other hand, generated higher levels of 8-oxo-G (11.5-fold) and FapyG (46-fold) for rRNA. On the other hand, ribosome samples did not show noticeable level of oxidation products with Fenton reaction. Gh levels were not significantly different between ribosome and rRNA. Observation of lower levels of oxidative lesions for ribosomes suggest that protein association could protect rRNA against ROS generated by the Fenton reaction. However, such protection was not observed for rRNA devoid of proteins (Figure 1B). Overall, the type of oxidation process and protein association status would determine the nature and amount of guanosine oxidation products in this experimental system.

**FIGURE 2:**
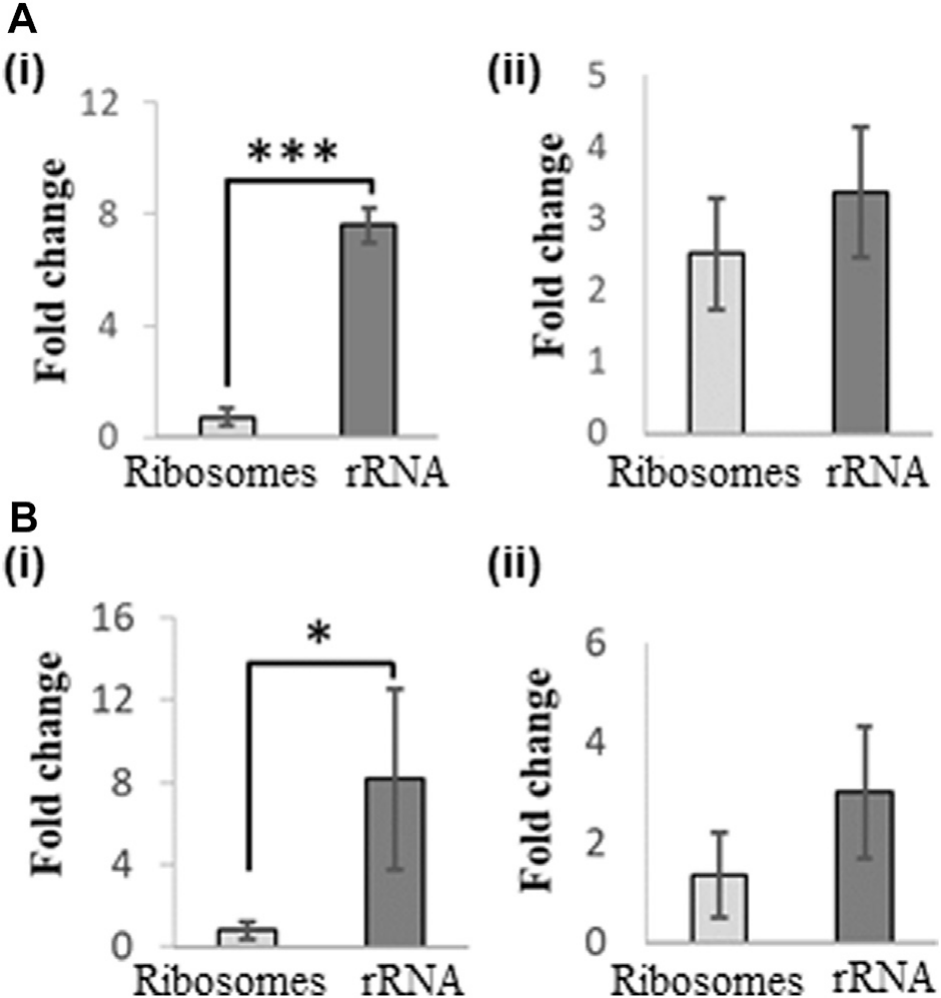
Changes in levels of adenosine oxidation products in rRNA following exposure of ribosome and rRNA to oxidative stress. **(A)** UVA-induced changes in levels of (i) 8-oxo-A, and (ii) FapyA. **(B)** Fenton-reaction induced changes in levels of (i) 8oxo-A, and (ii) FapyA. Fold change was expressed as the ratio of signal observed between exposed and unexposed samples following normalization against internal standard 5′bromo-2′deoxycytidine. Data are shown as the mean of five independent samples ±SD. Statistical differences were determined using unpaired two-tailed *t*-test (*p < 0.05; ***p < 0.001).

*Adenosine oxidation products*: Either UVA or Fenton reaction induced oxidative stress generated 8-oxo-A and FapyA (Supplementary Figure S3) for ribosomes and rRNA. However, 8-oxo-A was significantly higher for rRNA (8-fold) compared to ribosomes for both types of oxidative stress, but FapyA did not show any significant differences whether or not RNA bound with protein (Figure 2).

### 3.2 Effects of Oxidative Stress on rRNA Upon Cellular Exposure

#### 3.2.1 Oxidation and Fragmentation of rRNA Following Cellular Exposure to Photooxidative Stress

**FIGURE 3:**
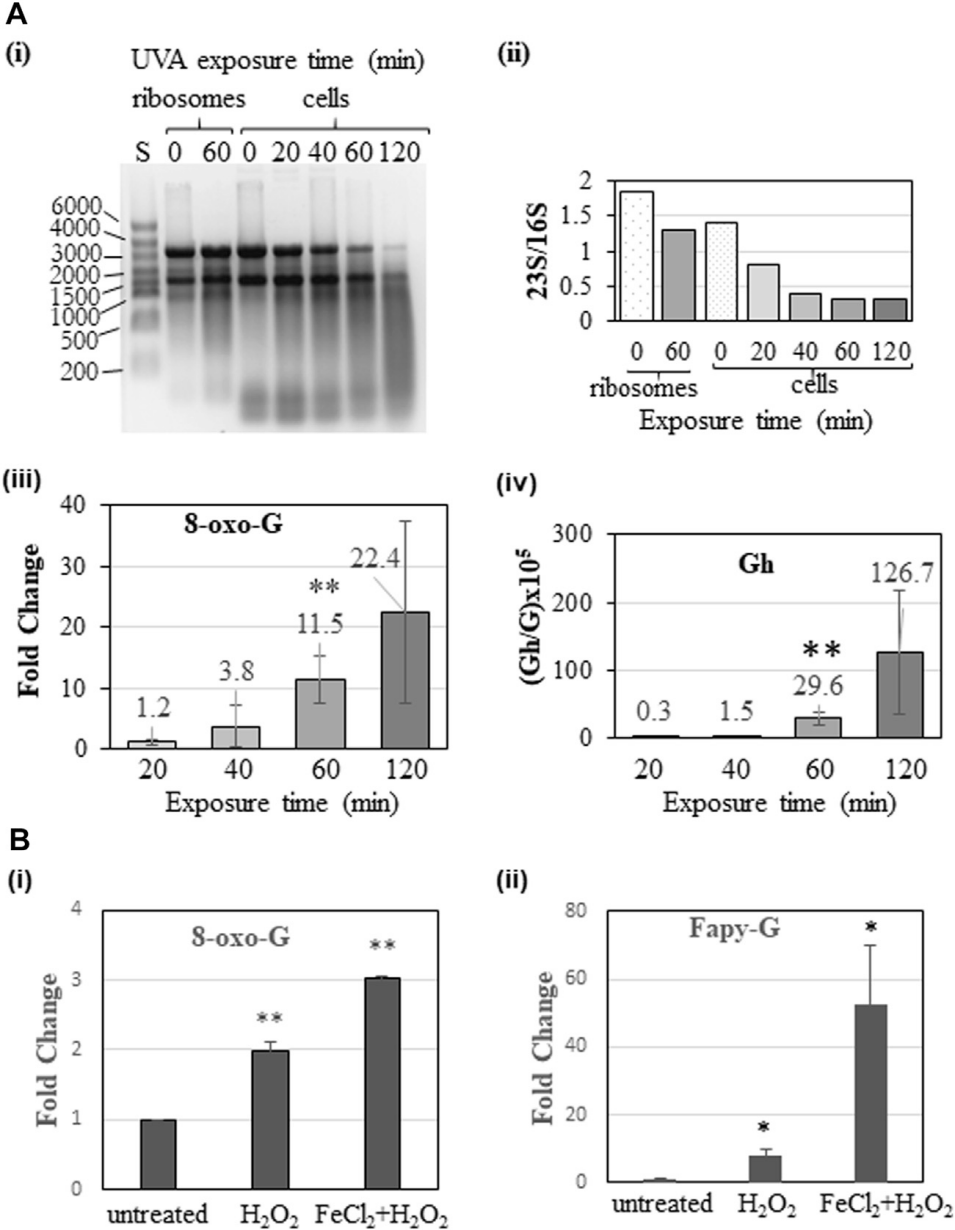
Effects of oxidative stress on rRNA upon cellular exposure. **(A)** UVA-induced oxidation and fragmentation of ribosomal RNA in *E. coli* cells. Ribosomes or mid-log phase (∼0.5 OD600) *E. coli* cell culture was exposed to UVA and fractionated ribosomal RNA was analyzed. (i) Gel electrophoretic analysis of ribosomal RNA (2 µg each). Lane S-size standards, number of nucleotides for each size standard RNA is denoted on left side. The lanes adjacent to the size standards contained rRNA isolated from ribosome following 0 (unexposed) and 60 min of exposure. Remaining lanes depict the rRNA isolated from unexposed (0) and exposed cells after 20, 40, 60, and 120 min of UVA exposure. (ii) Changes in levels of rRNA as measured by the ratio of respective signals. Elevated oxidation of guanosines as 8-oxo-G (iii) and Gh (iv) in rRNA with an increased UVA exposure are shown. **(B)** Fenton reaction induced oxidation in *E. coli* rRNA. *E. coli* cells at mid-log phase were treated with water (untreated) or 20 mM H_2_O_2_ or 10 µM FeCl_2_ and 20 mM H_2_O_2_ for 30 min and RNA extracted and analyzed. Elevated oxidation products of guanosine as 8-oxo-G (i) and FapyG (ii) are shown. Statistical significance was computed using unpaired two-tailed t-test (**p < 0.01, *p < 0.05).

Documenting the oxidation product levels of rRNA in the protein-bound and protein-free condition revealed the extent of increased susceptibility to ROS, when RNA is not bound with proteins. In order to understand the photooxidative effects under physiologically relevant conditions, K12 strain of *E. coli* was exposed to UVA for defined duration (20, 40, 60, and 120 min) and the rRNA analyzed for its integrity and oxidation products after LiCl-based fractionation. Gel electrophoresis of rRNA revealed elevated degradation of rRNA with increased exposure (Figure 3A) as reflected by the decreased signal intensity and ratio of rRNA (23S and 16S) soon after 40 min of exposure. Not surprisingly, the 8-oxo-G and Gh levels increased with increased duration of UVA exposure. Exposure of cells for 2 h led to RNA fragmentation, which presumably led to poor recovery and lower precision of the measured values of oxidation products for this exposure time. However, 1 h exposure yielded highest precision for oxidized nucleoside levels. This time point was used for further studies.

#### 3.2.2 Fenton Reaction-Induced Oxidation of Cellular rRNA

When cells were exposed to increasing levels of H_2_O_2_, significantly higher 8-oxo-G was observed from 5 mM onward, while Fapy-G required 20 mM concentration (Supplemental Figure S4). On the other hand, a combination of 10 µM FeCl_2_ and 20 mM H_2_O_2_ induced further increase in 8-oxo-G and Fapy G (Figure 3B) suggesting that the divalent ions such as Fe^++^ aggravate the oxidative stress in cellular system.

### 3.3 Loss of Enzymatic Post-transcriptional Modifications Increases Ribonucleic Acid Susceptibility to Reactive Oxygen Species

**FIGURE 4:**
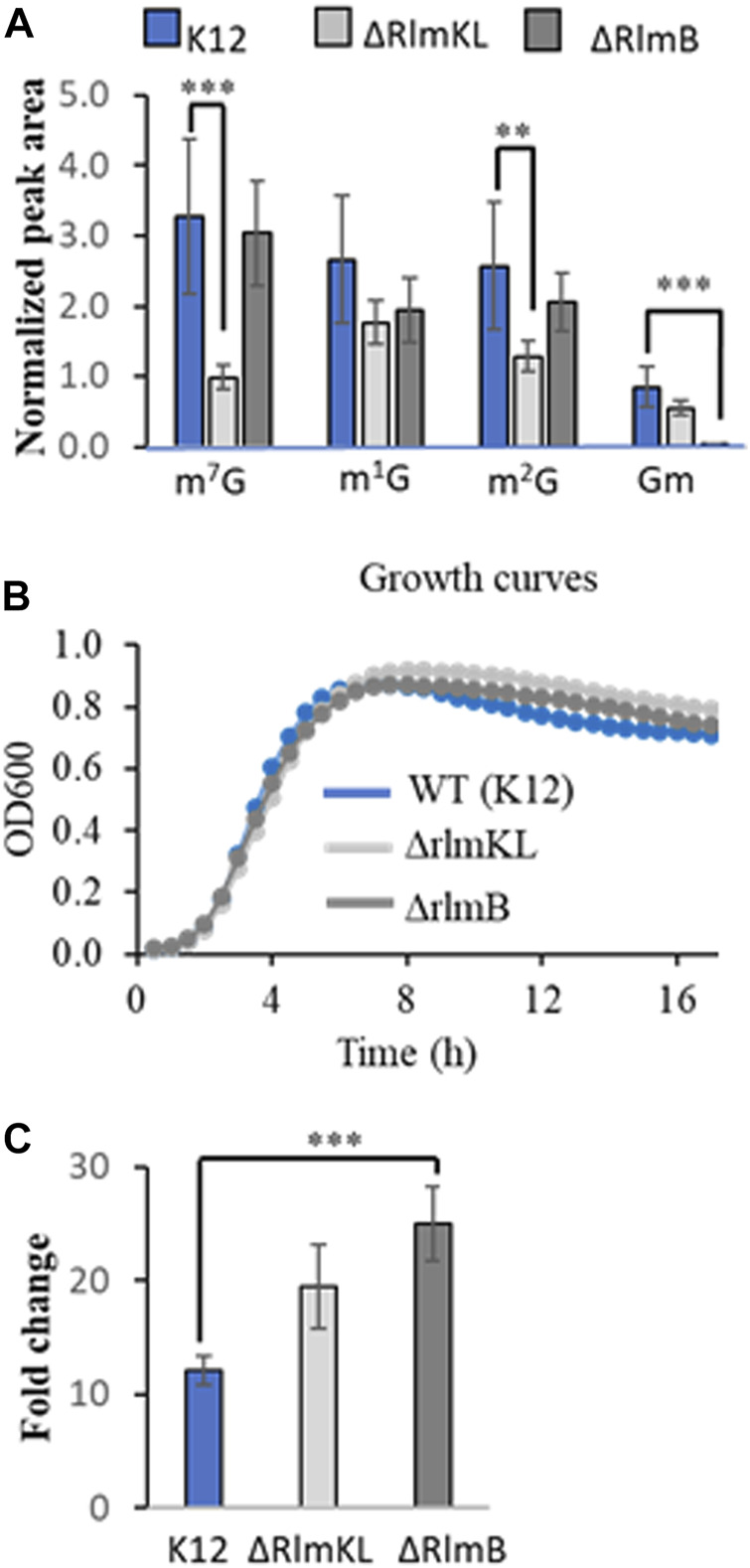
Altered levels of post-transcriptional (enzymatic or oxidative) modifications in rRNA isolated from ribosomes of K12 (wild Type), Δ*RlmKL* and Δ*RlmB*, *E. coli* strains. **(A)** Decreased levels of guanosine methylations, 7-methylguanosine (m^7^G), 2-methylguanosine (m^2^G) and 2′O-methylguanosine (Gm) in rRNA of *E. coli* strains deficient in respective methyltransferases. Peak area of each modification was normalized and plotted. Note the insignificant changes in 1-methylguanosine (m^1^G) levels in these strains. **(B)** Identical growth behavior of three different strains of *E. coli*. **C.** Fold change of 8oxo-G in rRNA isolated from UVA-exposed ribosomes of *E. coli* strains. Data is shown as the mean of three biological replicate samples ±SD after normalization against internal standard 5′bromo-2′deoxycytidine. Statistical significance was computed using unpaired two-tailed *t*-test (***p < 0.001, **p < 0.01).

To understand whether the absence of structure-stabilizing methylation modifications (Decatur and Fournier, 2002; Sergiev et al., 2011) influence the susceptibility of rRNA to ROS, we exposed the ribosomes derived from *E. coli* strains, K12, Δ*rlmKL* (exhibit loss of 7-methylguanine (m^7^G) at position 2069 and 2-methylguanine (m^2^G) at position 2,445) and Δ*rlmB* (exhibit loss of guanosine ribose methylation at position 2,251 of 23S rRNA) that are deficient in respective methyl transferases. Although these strains exhibited significantly lower levels of corresponding methylations in the ribosomal RNA (Figure 4A), the cells exhibited more or less identical growth behavior suggesting that the effects of undermethylated rRNA are minimal or insignificant (Figure 4B) under the tested culture conditions. However, upon UVA exposure, the 8-oxo-G levels increased 25-fold in the Δr*lmB* ribosomes compared to K12 (12-fold) or Δ*rlmKL* (19-fold) suggesting that the loss of ribose methylations could make the ribosomes more susceptible to ROS damage (Figure 4C).

**FIGURE 5:**
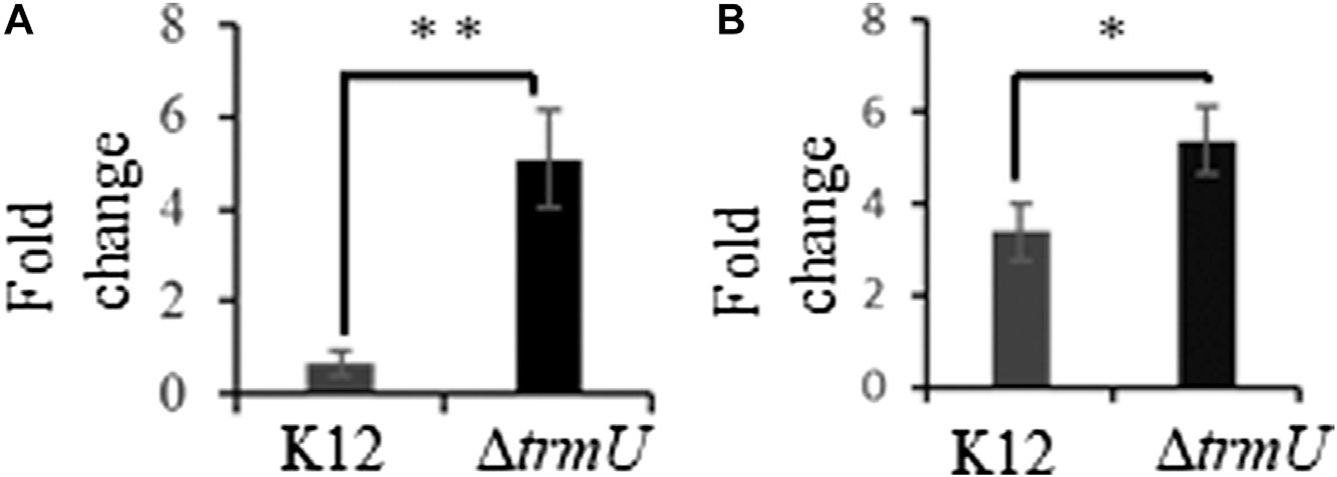
Fold change of 8-oxo-rG in transfer RNA **(A)** and total RNA **(B)** following exposure of K12 and Δ*trmU* strains of *E. coli* to Fenton reaction-induced oxidative stress. Data are shown as the mean of 3-5 biological replicates samples ±SD. Statistical differences were determined using unpaired two-tailed *t*-test (*p < 0.05; **p < 0.01).

The wobble modification in tRNA, mnm^5^s^2^U, is susceptible to UVA-induced oxidative degradation and the Δ*trmU* strain of *E. coli* deficient in this modification exhibited elevated levels of oxidized guanosine in tRNA upon exposure to UVA (Sun et al., 2018). We tested the possibility of such behavior under Fenton-reaction induced oxidative stress conditions. Exposure of this strain to the Fenton reaction caused higher levels of 8-oxo-G in tRNA consistent with previous observations, and such elevated levels were also reflected in total RNA level in comparison to the K-12 wildtype cells. (Figure 5).

## 4 Discussion

Besides transient association with proteins, cellular RNA is enzymatically modified with diverse chemical groups. To understand how such association and modification presence influence the RNA damage, we subjected the RNA to two types of oxidative stress and analyzed for the generation of advanced oxidation products apart from 8-oxo-G through LC-MS analysis. Our studies demonstrate that the kind of oxidative damage to RNA varies with the type of oxidative stress and the status of protein association. Formation of advanced oxidation products vary with the type of stress such as photooxidation or the Fenton reaction. This is because of the differences in half-lives of reactive oxygen species that range from nanosecond (hydroxyl radicals) or microsecond (superoxide anion, singlet oxygen and alkoxyl radical) to minutes (hydrogen peroxide, organic hydroperoxides) (Kehrer, 2000). RNA association with proteins significantly reduced advanced oxidation products in either type of oxidative stress. Another layer of complexity in the oxidative stress effects is the presence or absence of specific nucleoside modification that influence the oxidation levels in RNA.

### 4.1 Type of Oxidative Stress Determines the Oxidative Damage

Consistent with the previous reports (Liu et al., 2012), the basal levels of 8-oxo-G exhibited wide variation depending on the additives included in the samples even though all the precautions (dark incubation, minimal exposure to air) were followed. Iron chloride addition alone increased the basal levels of 8-oxo-G (Supplemental Figure S1) presumably due to spurious oxidation during sample handling and/or LC-MS analysis (Gedik and Collins, 2005). However, addition of oxidant such as hydrogen peroxide, or exposure to UVA increased the oxidation of purines up to 7-fold for 8-oxo-G, and 25 Gh lesions or 46 FapyG lesions per 10^5^ guanosines (Figure 1) compared to riboflavin or iron chloride added samples (Supplemental Figures S2, S3).

Exposure of rRNA and ribosome yielded 8-oxo-G and advanced oxidation products, Gh or FapyG. Exposure to H_2_O_2_ at lower concentration (1–10 mM) did not cause significant levels of advanced oxidation products (other than 8-oxo-G) (data not shown). Higher concentrations of H_2_O_2_ (20 mM) in combination with iron chloride (induced Fenton reaction) caused higher levels of advanced oxidation products. However, Gh formation was predominantly noticed in UVA exposure and FapyG by the Fenton reaction. These observations indicate the prevalence of specific oxidation pathways depending on photooxidation or chemical oxidation. The predominant type of ROS generated, for example, singlet oxygen by UVA (Miyamoto et al., 2003; Baier et al., 2007; Schuch et al., 2013) and hydroxyl radicals by Fenton reaction (Kehrer, 2000; Koppenol, 2001) could be one reason for operation of alternate oxidation pathways.

### 4.2 Association of Ribonucleic Acid With Proteins Reduce the Levels of Advanced Oxidation Products

Ribosomal protein association with rRNA, in general, decreased the levels of advanced oxidation (further oxidation of 8-oxo-G) products for UVA exposure (Figure 1). There was general decrease in purine oxidation levels when ribosomes were exposed to the Fenton reaction conditions (Figure 2). Ribosomal proteins are located in the outer shell of ribosome, which provides an easier solvent-accessibility for ROS (Nikolay et al., 2015). They are also known for maintaining ribosomes’ structure, suggesting that their absence alters ribosome conformation and may expose rRNA nucleotides previously “sheltered” in the inner core. Aside from their localization and function, ribosomal proteins are composed of amino acids that have a lower calculated reduction potential compared to rRNA nucleotides, ranging from 0.85 V (tryptophan) to 1.67 V (lysine) (Close and Wardman, 2018). Therefore, the absence of ribosomal proteins could lead to enhanced oxidative damage to rRNA. This raises interesting questions about the role of proteins in influencing the oxidation. Do they need to be bound to the rRNA for reducing the damage or unrelated proteins or free amino acids could also influence extent of damage? Future studies involving RNA and RNA binding proteins or mixtures of RNA and proteins or amino acids could provide more insight into the specific or non-specific nature of these effects. Nevertheless, these observations clearly indicate that the chance of aggravated oxidative damage is higher if the RNA is free of proteins. Locating the oxidation sites with or without protein presence could also help answer this query. Increased fragmentation of oxidized RNA in the cellular conditions might be due to the higher affinity of oxidized RNA to enzymes such as polynucleotide phosphorylase leading to RNA degradation (Hayakawa et al., 2001; Malla and Li, 2019).

### 4.3 Lack of Enzymatic Ribonucleoside Modification Could Act as Agonist for Oxidative Stress

We evaluated the impact of oxidative stress on ribosomes when the cells are deficient in specific methyltransferases. These strains of *E. coli* (*ΔrlmKL, ΔrlmB*) did not show any significant differences in their growth behavior under normal cell culture conditions. The methylations catalyzed by these enzymes are located in Domain V of 23S rRNA. The ribose methylation, Gm is located at position 2,251 in the peptidyl transferase center (PTC) and is a universally conserved modification (Polikanov et al., 2015). Modifications, m^7^G (at 2069) and m^2^G (at 2,445) are installed by a unique dual fused methyltransferase RlmKL (Kimura et al., 2012). UVA exposure of the ribosomes isolated from these strains showed elevated 8-oxo-G levels. While the increase in 8-oxo-G levels is not significant (*p* value = 0.053) for *ΔrlmKL,* those of *ΔrlmB* strain exhibited significantly higher levels than K12 cells (*p* = 0.00064). It is interesting to note that although RlmKL installs methyl groups at two different positions (2069 and 2,445) of 23S rRNA compared to RlmB (at position 2,251) (Popova and Williamson, 2014; Sergiev et al., 2018), the latter strain seems to be more susceptible to oxidative stress (Figure 4). Further studies involving modification mapping experiments (Sun et al., 2020; Thakur et al., 2020) could identify the exact locations in the sequence that might serve as hotspots for oxidative damage. Nevertheless, these studies indicate that although some post-transcriptional methylations are not essential for normal growth, their absence could aggravate the vulnerability of rRNA to oxidative damage. This is presumably due to the instability of the folded structure, because methylations on the nitrogenous base and ribose play an important role in stabilizing the structure (Decatur and Fournier, 2002; Sergiev et al., 2011) and the interactions with other translational apparatus (Polikanov et al., 2015). Such vulnerability is different from that of the oxidative degradation of post-transcriptional modifications containing -amino, -oxy, and sulfur groups. This is because methylations, in general, were found to be less susceptible to oxidative degradation in our previous studies (Sun et al., 2018).

Similarly, the absence of thiolation in mnm^5^s^2^U also led to increased guanosine oxidation upon UVA exposure in the enzyme-deficient cells (Δ*trmU*) (Sun et al., 2018). This observation is recapitulated in the current investigations for transfer RNA and even extended to total RNA with Fenton reaction studies, where the 8-oxo-G levels significantly increased in the mutant cells (Figure 5). These observations strongly suggest that certain nucleoside modifications are associated with oxidative stress effects. Recently, *E. coli* cells deficient in mnm^5^s^2^U are shown to be highly sensitive to hydrogen peroxide and mnm^5^s^2^U is required for stress-resistance responses (Jingjing et al., 2021) suggesting that the observed effects in our studies are not coincidental. Overall, these studies strongly indicate that ribosomal proteins and post-transcriptional nucleoside modifications could play multiple roles in not only maintaining optimal structure and decoding function but also protect against the adverse effects of oxidative stress to perform optimal mRNA translation in the cell.

In summary, the pattern of oxidative damage to RNA (8-oxo-G and its advanced oxidation products) is altered by the protein association and presence of post-transcriptional nucleoside modifications. RNA oxidation and degradation levels under cellular condition were higher compared to the exposure of ribosomes. Although, loss of methyl groups at two different positions did not increase the susceptibility, absence of ribose methylation at another position increased the susceptibility of rRNA to oxidative damage in ribosomes compared to that of wild type *E. coli* suggesting a position-specific dependence. Similarly, deficiency of ROS-susceptible nucleoside modification increased the overall oxidative damage in tRNA, and total RNA compared to wild type strain. This suggests that it is not the number, but the nature and status of modification at a defined position that determines the overall susceptibility of RNA to ROS.

## Data Availability

The raw data supporting the conclusion of this article will be made available by the authors, without undue reservation.
