# Supplementary figures and images for: Oxidative Damage to RNA is Altered by the Presence of Interacting Proteins or Modified Nucleosides
Source: Front Mol Biosci. 2021 Jul 1;8:697149. doi: 10.3389/fmolb.2021.697149 (PMC8281250; doi:10.3389/fmolb.2021.697149)

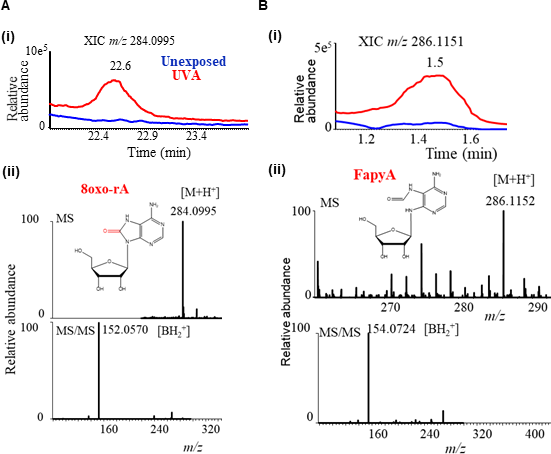

Supplement: Supplementary file 1 [file Image3.TIF]

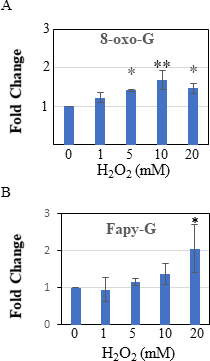

Supplement: Supplementary file 2 [file Image4.TIF]

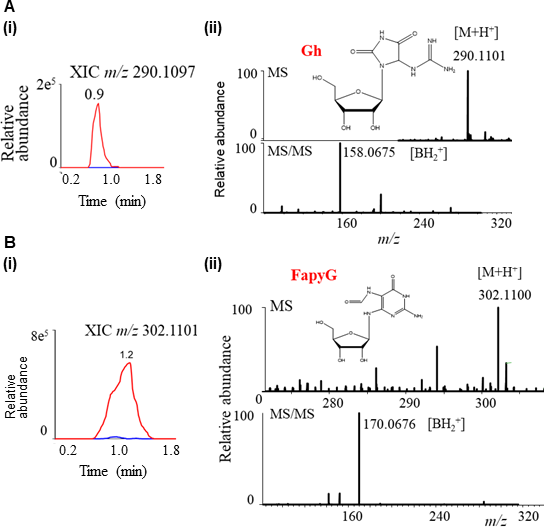

Supplement: Supplementary file 3 [file Image2.TIF]

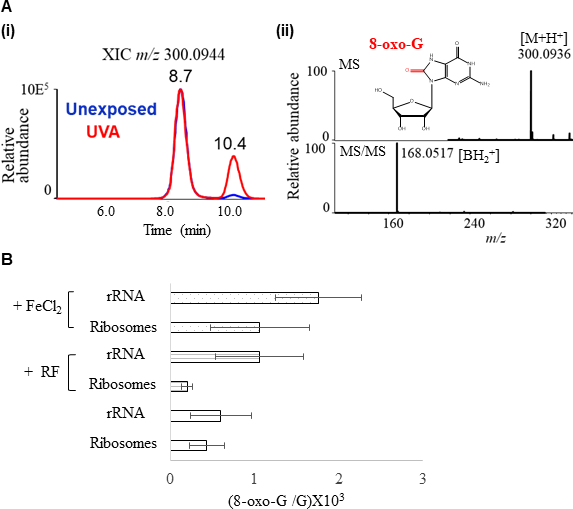

Supplement: Supplementary file 4 [file Image1.TIF]
