# Supplementary material for: Oxidative Damage to RNA is Altered by the Presence of Interacting Proteins or Modified Nucleosides
Source: Front Mol Biosci. 2021 Jul 1;8:697149. doi: 10.3389/fmolb.2021.697149 (PMC8281250; doi:10.3389/fmolb.2021.697149)
Supplement: Supplementary file 5 [file DataSheet1.PDF]

Supplementary Material

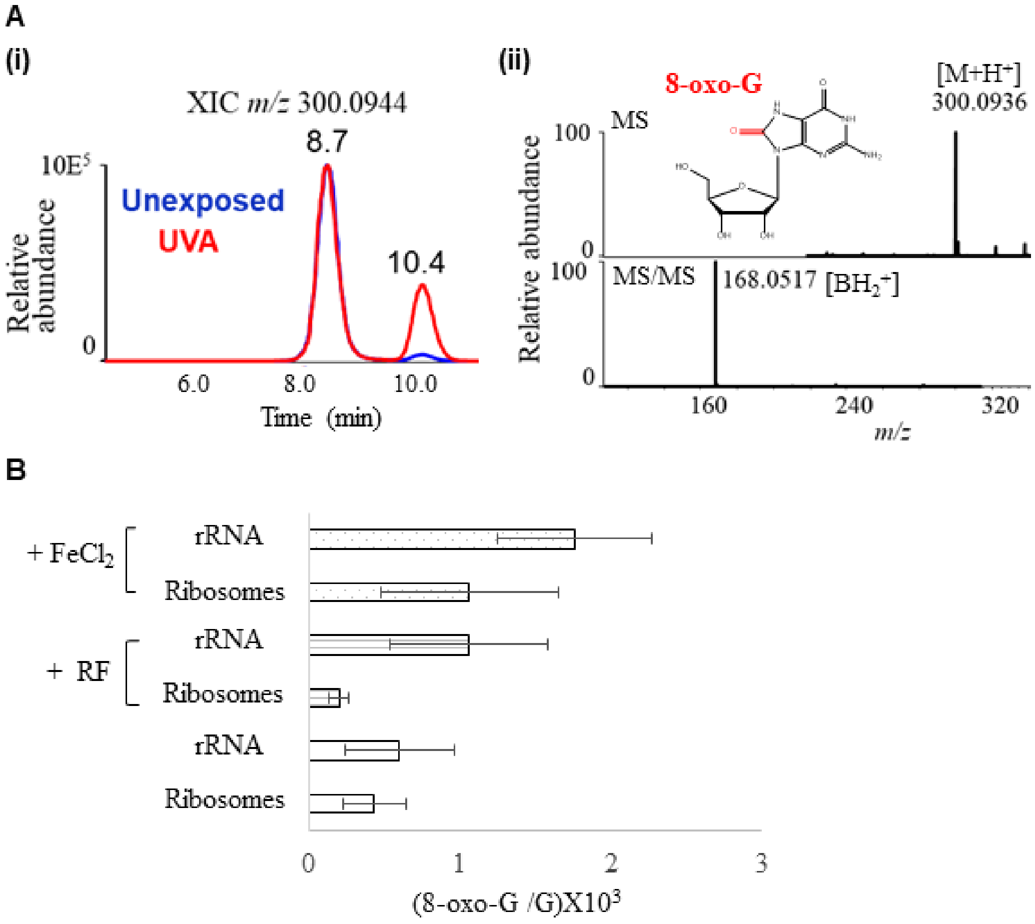

Supplementary Figure 1

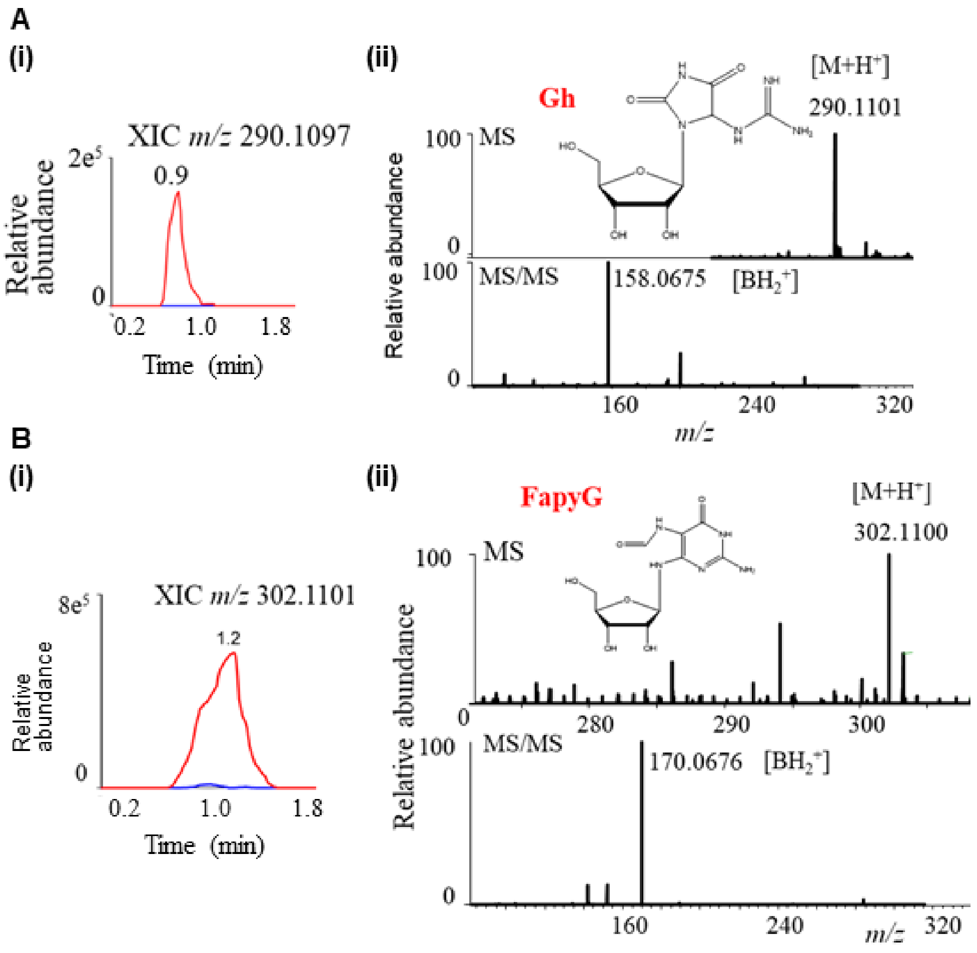

Supplementary Figure 2

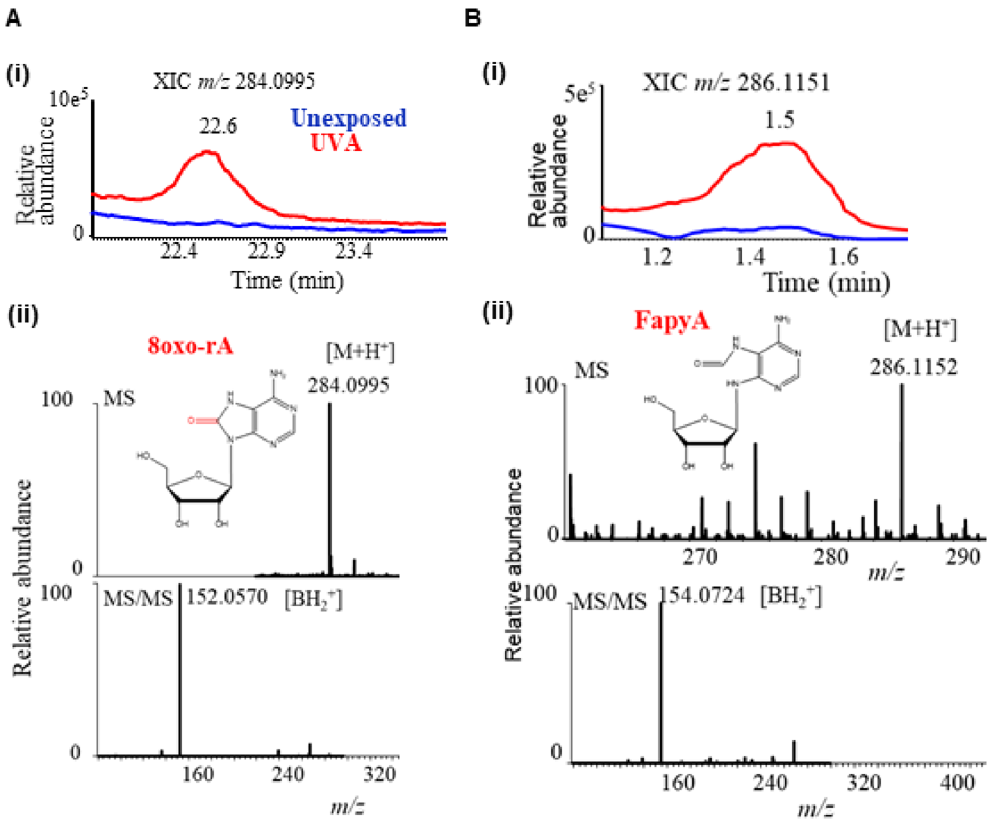

Supplementary Figure 3

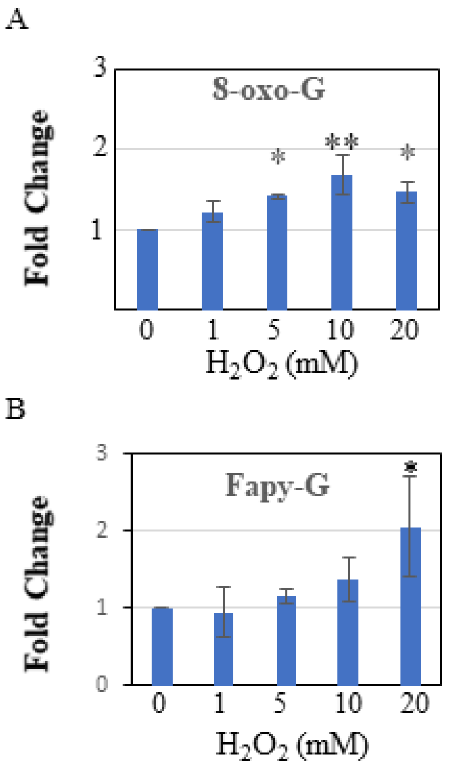

Supplementary Figure 4

Supplementary Figure Legends

Supplemental Figure 1: LC-MS based detection of guanosine oxidation. A. Detection of 8-oxo-rG. (i) Extracted ion chromatogram (XIC) for 8-oxo-G (*m/z* 300.0944). The 8-oxo-G resident in RNA exhibits longer retention time (10.4 min) compared to electrospray generated oxidation signal (8.7 min). Note the near identical alignment of electrospray-generated oxidized guanosine signal for both unexposed (but contained RF or iron chloride, blue color trace) and exposed samples (red trace) at 8.7 min but not at 10.4 min. (ii) Mass spectra of XIC. Top panel depicts the molecular ion ($M+H^+$) in the mass spectrum (MS) of XIC and the bottom panel shows the signal for 8-oxo-guanine nucleobase (BH_2^+, m/z 168.0517) in the tandem mass spectrum (MS/MS) following loss of ribose sugar (132 units) due to collision-induced dissociation of molecular ion. B. : Comparison of the base line levels of 8-oxo-G in rRNA. Equivalent amounts of rRNA or ribosome (0.12 μg μL^{-1}) was incubated with either $FeCl_2$ (10 μM) or riboflavin (RF, 100 μM) or water for 1 h and processed for nucleoside analysis as described in Materials and Methods. The quantum of signal for 8-oxo-G was normalized to get an average number of lesions for 1000 guanosine residues in ribosomal RNA

Supplemental Figure 2: LC-MS based detection of guanosine oxidation products. A. Detection of Gh in rRNA. (i) XIC for Gh (*m/z* 290.1097) is shown. (ii) Mass spectra of the molecular ion (top panel) and the nucleobase ion (bottom panel) of the XIC at 0.9 min are shown. B. Detection of FapyG in rRNA. (i) XIC for FapyG (*m/z* 302.1101) is depicted. (ii) Mass spectra of molecular ion (top panel) and nucleobase ion (bottom panel) of the XIC at 1.2 min are shown.

Supplementary Figure 3: LC-MS based detection of adenosine oxidation products. A. Detection of 8-oxo-A. (i) Extracted ion chromatogram (XIC) for 8-oxo-A (*m/z* 284.0995) is shown. (ii) Mass spectra of XIC at 22.6 min. Top panel depicts the molecular ion ($M+H^+$) in the mass spectrum (MS) of XIC and the bottom panel shows the signal for 8-oxo-adenine nucleobase (BH_2^+, m/z 152.0570) in the tandem mass spectrum (MS/MS). (B) Detection of FapyA in rRNA. (i) XIC for FapyA (m/z 286.1151). (ii) Mass spectra of molecular ion (top panel) and nucleobase ion (bottom panel) corresponding to the XIC at 1.5 min are shown.

Supplementary Figure 4: Hydrogen peroxide (H_2O_2) induced oxidation of guanosine in rRNA of E. coli. Cells at mid-log phase were exposed to indicated concentration of H_2O_2 , rRNA extracted and analyzed for guanosine oxidation products, 8-oxo-G (A) and Fapy-G (B).
